# Liquid Crystal Networks on Thermoplastics: Reprogrammable Photo‐Responsive Actuators

**DOI:** 10.1002/anie.201915147

**Published:** 2020-01-30

**Authors:** Rob C. P. Verpaalen, Marina Pilz da Cunha, Tom A. P. Engels, Michael G. Debije, Albert P. H. J. Schenning

**Affiliations:** ^1^ Eindhoven University of Technology, Chemical Engineering & Chemistry Functional Organic Materials & Devices (SFD) Helix building STO 0.34 Den Dolech 2, 5612 AZ Eindhoven The Netherlands; ^2^ Technische Universiteit Eindhoven Faculteit Werktuigbouwkunde The Netherlands; ^3^ Technische Universiteit Eindhoven Faculteit Scheikundige Technologie The Netherlands

**Keywords:** liquid crystals, photo-responsive materials, polymers, reprogrammability, thermoplastic

## Abstract

Arbitrary shape (re)programming is appealing for fabricating untethered shape‐morphing photo‐actuators with intricate configurations and features. We present re‐programmable light‐responsive thermoplastic actuators with arbitrary initial shapes through spray‐coating of polyethylene terephthalate (PET) with an azobenzene‐doped light‐responsive liquid crystal network (LCN). The initial geometry of the actuator is controlled by thermally shaping and fixing the thermoplastic PET, allowing arbitrary shapes, including origami‐like folds and left‐ and right‐handed helicity within a single sample. The thermally fixed geometries can be reversibly actuated through light exposure, with fast, reversible area‐specific actuation such as winding, unwinding and unfolding. By shape re‐programming, the same sample can be re‐designed and light‐actuated again. The strategy presented here demonstrates easy fabrication of mechanically robust, recyclable, photo‐responsive actuators with highly tuneable geometries and actuation modes.

## Graphical abstract

**Thermoplastic polyethylene terephthalate** (PET) were spray‐coated with an azobenzene‐doped liquid‐crystal network (LCN). Arbitrary shapes are programmed by thermal shaping and fixing of PET, enabling untethered, area‐specific light response with origami‐like folds. The thermoplastic actuator demonstrates multi‐modal shape morphing in a single sample and can be re‐designed and light‐actuated repeatedly.

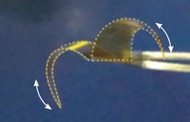

## Introduction

**Figure 1:**
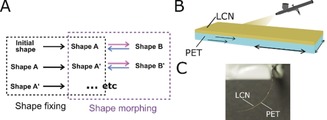
The reconfigurable light‐responsive thermoplastic actuator. A) Mechanism for a reconfigurable shape fixing and shape morphing actuator. The shape fixing of thermoplastic polymer enable the programming of an arbitrary moulded initial shape (shape A). Application of light as a stimulus causes reversible shape morphing of the programmed geometry (between shape A and shape B). Reprogramming of the moulded initial shape allows for any new shape morphing (between shape A′ and shape B′) B) Schematic depiction of the PET/LCN bilayer, with PET serving as the thermoplastic polymer and the spray‐coated LCN as the light‐responsive polymer. C) Photograph of a light‐responsive thermoplastic actuator with dimensions 20×3×0.016 mm^3^. The actuator has a pre‐bent shape immediately after fabrication in which the LCN coating is always on the inside of the slight curl.

The last decade has seen an increasing development of stimuli‐responsive soft materials capable of complex, programmed shape changes.^1^, ^2^, ^3^ Wireless control of shape changing materials with rapid reversible motions has paved the way towards untethered and small‐scale walking soft robots and muscle‐like actuators.^4^ The ease of addressability and spatial‐temporal control achieved with light has made it a preferred stimulus for polymeric actuators and soft robots.^4^, ^5^ However, unlike natural systems which have complex geometrical shapes and multiple actuation modes, synthetic light‐driven soft actuators are often limited in their starting geometry, being usually flat, inhibiting the development of intricate architectures, and being primarily restricted to just one mode of actuation. In addition, synthetic actuators generally lack shape re‐programming capability owning to the inability to reprogram the deformation.^6^ Developing actuators that allow for shape moulding to any desired initial shape (shape A in Figure 1 A), consecutive reversible light‐responsive actuation, shape morphing (shape A to B), with possible re‐programmability (shape A to A′) and multi‐modal movement, will greatly increase the potential of photo‐responsive materials alleviating present restrictions in actuation design and freedom.

Thermoplastic polymers can be moulded and re‐moulded when heated, allowing the preparation of any desired permanent shape. The mechanical robustness and ductility of thermoplastic polymers, such as polyethylene terephthalate (PET), for example, allows for its deformation into origami‐like shapes with sharp folds.^7^ This makes thermoplastic materials attractive as a highly versatile polymer for shape fixing of light‐driven soft actuators. Azobenzene doped liquid crystalline networks are a prominent class of materials for photo‐driven actuators, demonstrating fast and reversible shape changes.^8^, ^9^, ^10^, ^11^ The tuneable molecular architecture of LCNs prior to polymerization allows for programmed actuation such as bending, twisting or rolling. Multi‐modal shape morphing such as helical winding and unwinding within single liquid crystal (LC) actuators has been realized by employing different cutting directions,^12^, ^13^ dual‐layer actuators with contrasting orientation directions,^14^ alignment control through directional printing^15^ or patterning.^16^, ^17^, ^18^, ^19^ However, in these examples, the covalent crosslinked nature of the network prevents recyclable shape programming. LC‐based actuators usually have a set initial shape determined by the fabrication conditions. Recently, 3D printing has allowed for the construction of LC‐based actuators having pre‐designed initial shapes.^20^, ^21^, ^22^ The ability to shape fix the starting geometry into any arbitrary shape and subsequently trigger reversible actuation through exposure to light, as done for temperature‐driven actuators through shape memory or by using dynamic covalent bonds,^23^, ^24^, ^25^, ^26^, ^27^, ^28^, ^29^, ^30^, ^31^ remains largely unexplored. Light encoding has been employed by Priimagi and co‐workers as an approach towards photo‐rewritable programming of light‐driven actuation in LCNs.^32^ Yet, even such innovative developments do not alleviate the restrictions of the flat starting geometry of the actuators. Ikeda and co‐workers demonstrated an LC‐based photo actuator with control over the initial actuator shape as well as reversible light‐driven actuation, but recyclable shape programming was not demonstrated.^33^

We now present a strategy for creating mechanically robust, recyclable shape programmable light‐driven actuators with pre‐designed initial geometries which can rapidly actuate upon exposure to light with multi‐modal motions. In this method, shape fixing and actuation are decoupled by using independent stimuli, allowing for greater design versatility and control over the initial geometry of the photo actuator. The actuators have a bilayer architecture consisting of PET spray‐coated with a light‐responsive LCN, Figure 1 B and C. The PET has multiple roles: acting as an aligning layer for the spray‐coated LCN,^34^, ^35^ giving the actuator mechanical robustness, and acting as a shape programming thermoplastic polymer to create a pre‐designed initial geometry, which can be triggered by light to perform shape‐dependent actuation in which the motion is encoded in the geometrical design. Through recyclable shape programming, the same sample can be re‐designed (shape A to A′, Figure 1) from origami‐like folded geometries into more classical bent or helical actuators, for example. Our strategy opens doors towards a facile method for functionalization of thermoplastic polymers and offers unprecedented freedom for creating light‐responsive actuators with high degree of robustness.

## Results and Discussion

The thermoplastic actuator was fabricated using a scalable spray‐coating method, depositing the light‐responsive LCs from xylene on a biaxially stretched 12 μm thick PET polymer (Figure S1 in the Supporting Information).^36^, ^37^, ^38^ The sprayed nematic LC mixture (Figure S2) self‐aligned in a splay molecular alignment on the PET, see Figure S3.^37^ The LC mixture was photopolymerized in the nematic phase at 85 °C for 10 minutes in an inert nitrogen atmosphere (Figure S4). To eliminate interfacial polymerization‐induced stresses at the PET‐LCN interface, the bilayer strip was post‐cured at 130 °C (*T*>*T*_g PET_, *T*_g PET_≈120 °C, see below) and cooled to room temperature. The bilayers were cut with the LC molecular director parallel to the strip's long axis (20×3×0.016 mm^3^). The expansion of both the 4 μm thick LCN and 12 μm thick PET layers coupled with an overall increase in LCN order upon cooling from the polymerization temperature give rise to a small pre‐bend of the bilayer strip (Figure 1 C).

**Figure 2:**
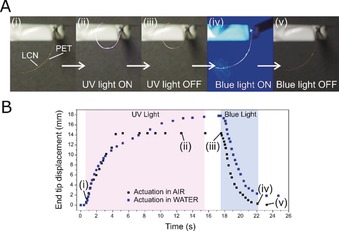
Light‐driven actuation of the LCN‐PET actuator with dimensions 20×3×0.016 mm^3^ A) Snapshots of actuation in air: (i) the pre‐bent strip at room temperature prior any illumination; (ii) actuator exposed to UV light (170 mW cm^−2^); (iii) actuator after removal of UV irradiation; (iv) blue light (300 mW cm^−2^) triggers return to straighter geometry; (v) turning off the blue light results in no noticeable shape changes. The collimated light source is incident from the left. B) Plot depicting end tip displacement of the actuator in both water and in air as a function of time, during UV and blue light exposure, shown by the violet and blue boxes, respectively, and upon switching off the lights. The numbered insets in the plot refer to the corresponding figures in (A).

We first illustrate the light‐responsive reversible actuation of the bilayer through exposure to UV and blue light (365 and 455 nm, respectively; see Figure 2 A, Figure S5 and Movie S1). The macroscopic bending deformation of the bilayer is driven by the photoisomer's dimensional change from the extended *trans* configuration to the unstable *cis* isomer. Under UV illumination the azobenzene chromophores in the LCN isomerize, leading to an anisotropic contraction along the molecular axis, which rapidly bends the actuator (exceeding 5 mm s^−1^: see Figure 2). It is remarkable to observe that by depositing a 4 μm thin LCN, a 12 μm PET substrate was bent so easily attaining bending speeds similar to single layer >20 μm thick LCN films.^39^ Upon removal of the UV irradiation, the bilayer retains its bent state; this shape persists due to the slow (hours) back isomerization of the *cis* azobenzene isomer.^40^, ^41^ The macroscopic bending deformation of the pre‐bent bilayer in Figure 1 C is independent of the incident UV illumination direction, always resulting in the LCN on the interior of the bent strip. The direction of bending is commonly found in uniaxial aligned bilayer systems, indicating that actuation is dictated by the dominant planar section in the splay LCN. Mechanical relaxation can be immediately triggered by exposure to blue light, which promotes the azobenzene *cis‐trans* isomerization (Figure 2). Photo‐actuation cycles were repeated dozens of times without any visible fatigue of the bilayer. Illumination of an uncoated PET layer does not cause such macroscopic motion.

Tracking of the bilayer temperature during irradiation revealed the surface remains around 33 °C under 170 mW cm^−2^ UV irradiation (Figure S6). Hence, the actuation is primarily photomechanical in nature, driven by the exerted network pull‐effect derived from *cis* isomers, leading to an anisotropic contraction along the molecular axis.^40^, ^41^ When the actuator is submerged in water, the bilayer displays bending similar to the dry environment when illuminated, Movie S2. Since, the thermal contribution to the back isomerization of the azobenzene moieties (*cis–trans*) is absent, as water acts as a heat sink, this results in an overall higher *cis* isomer population, hence in a marginally larger actuation upon irradiation underwater.

**Figure 3:**
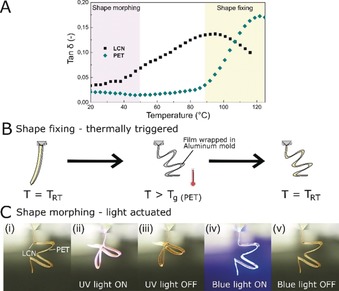
Actuator shape moulding. A) The damping ability, *tan* *δ*, of individual LCN and PET layers recorded as a function of temperature. The yellow box in the plot represents the temperature region in which thermal shape fixing of the bilayer is executed. The violet outline shows the temperature range for the light actuated shape morphing. B) Thermal shape fixing of LCN‐PET bilayer actuators. Bilayer strips are wrapped in aluminium foil, manually deformed and heated in PET's glass transition region (*T*≈100 °C). Upon removal from the oven, the strips retain the deformed shape. C) Photographic stills taken from the shape programmed bilayer actuator while being exposed to UV and blue light from the left.

Dynamic mechanical analysis (DMA) reveals that freestanding LCN and PET films have glass transition temperatures at approximately 90 and 120 °C, respectively (Figure 3 A), providing a temperature window in which easy shape fixing may be executed (Figure 1). For shape fixing, the bilayers were wrapped in aluminium foil to retain the designed shape, manually deformed and placed in an oven at *T*≈100 °C (i.e. PET's glass transition regime) for 15 minutes, Figure 3 B. At this temperature, the individual polymers operate in or close to their rubbery state. Removing the samples from the heat source and cooling to room temperature (*T*=*T_RT_*) results in thermal quenching, immediately fixing the programmed shape. Short thermal treatment (i.e. less than 30 seconds) have been found to be sufficient to fix the actuator into a desired shape. The mechanical robustness of the PET is crucial in the shape fixing step, increasing the ductility of the material when compared to single layer brittle LCN actuators, allowing for origami‐like shapes to be made, such as accordion‐like structures with sharp folds, Figure 3 C. This programmed shape can then be actuated by light and demonstrates reversible shape morphing from the accordion‐like geometry (Figure 3 C(i)) into a flower‐like shape folded shape (Figure 3 C(ii)) after UV light irradiation, Movie S3. As with the non‐moulded sample in Figure 2 A, the actuated shape persists after removal of UV irradiation, Figure 3 C(iii). Subsequent blue light exposure reverts the deformed film into the shape programmed accordion geometry seen in Figure 3 C(v) and not the original straight strip, (Figure 2 A(i)). The potential for facile shape programming allows for creative light‐driven origami‐like materials, such as folded actuator “birds” whose wings can be selectively actuated to bend resembling the motion of flight, Figure S7 and Movie S4.

**Figure 4:**
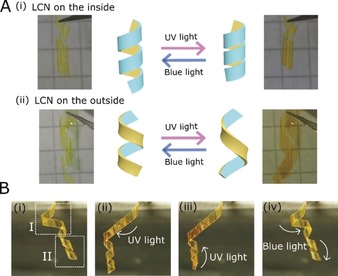
Reprogramming the actuation mode by shape configuration. A) Light actuation of spiral bilayer actuators with the LCN (i) on the inside of the helix and (ii) on the outside of the helix. In (i), UV light exposure results in helical winding and in (ii), it results in helical unwinding. Both deformations are reversible after sufficient time or through blue light illumination. B) Selective light response using a dual‐mode actuator composed of two sections. Section I has the LCN on the outside of the bilayer and section II on the inside.

We expand on the opportunities brought by the easy shape reconfiguration of the actuator by tuning the film geometry during a programming step: two identical helical shaped strips can be made to actuate in contrasting manners, Figure 4 A. By manually twisting the same bilayer strip with the LCN coating on either the inside Figure 4 A(i) or on the outside Figure 4 A(ii) of the helix results in one helix winding upon UV light illumination while the other helix unwinds. In both cases, blue light reversed the macroscopic deformation of the helices.

Our shape design method can also be used to attain multi‐modal actuation in single actuators by local light exposure. As an example, a single actuator is fabricated having two opposite helical twisted structures in the same strip, resulting in opposing actuation modes within a single sample, Figure 4 B. Multiple intermediately shaped morphing geometries are possible: selectively addressing the top of the two regions straightens the top curl (I) by *trans*‐to‐*cis* isomerization, while the lower spiral preserves its helical shape, Figure 4 B(i), and subsequent selective UV light exposure tightens the lower spiral (II), with the LCN on the inside of the helix, Figure 4 B(ii). Both actuation modes could be reversed together or independently using blue light. A more random programmed shape actuator demonstrates the true versatility of the shape fixing method, Figure S8. In this latter example, two sections of the actuator perform different actuation modes when triggered by UV light: the top unbending and the bottom curling. The spatial control obtained with focused light allows for area selective actuation, Movie S5.

**Figure 5:**
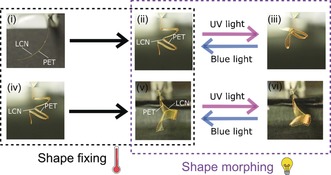
Demonstration of shape programming/re‐programming and shape morphing possibilities using a single actuator strip. Through thermal programming, a bilayer strip is shown to be moulded in two different geometrical shapes. Subsequent light actuation of the shapes results in diverse actuation in all different cases. The accordion‐like region performs origami‐like bending while the spiral region unwinds.

We further demonstrate actuator reprogramming by using the bilayer strip thermally moulded into an accordion‐like shape with sharp folds which is subsequently reconfigured into a completely different geometry such as a spiral, Figure 5. Light‐driven actuation shows the accordion‐like shape performing origami‐like bending ((ii) to (iii) in Figure 5) and the spiral helical region unwinding, ((v) to (vi) in Figure 5). The shape design and re‐design allows for versatility and adds significant functional advantages, allowing the same polymer film to be re‐used for the execution of diverse motions, distinguishing these actuators from existing devices.

## Conclusion

We have developed novel, light‐responsive thermoplastic actuators with versatility in shape design, re‐programmability, and multi‐modal actuation. Composed of a spray‐coated LCN on a stretched PET template, the bilayer shows rapid, reversible responses to light, offering excellent shape morphing capabilities. Compared to single LCN actuators, this bilayer approach offers the practical advantage of utilizing a scalable spray‐coating method, eliminating alignment layers, and requiring only thin 4 μm LCN coatings to achieve similar actuation to >20 μm thick films commonly employed. Thermoplastics can be used to shape fix any arbitrary starting geometry via a thermal treatment. More complex liquid crystalline alignments or selective patterning of the LCN onto the PET can be explored to expand on the range of light triggered actuation. Underwater actuation shows the bilayer's potential use in aqueous or amphibious soft robotics. This new generation of mouldable actuators offers endless possibilities for fabricating actuators with pre‐designed geometries in which the actuation mode can be engineered through a shape programming step, without the need for complex cutting steps or chemical patterning. Additionally, we envision that this facile fabrication method can serve to functionalize other thermoplastic polymers such as polyimides and polyamides. The exceptional versatility and ease of fabrication of our light‐responsive thermoplastic actuators establishes a new toolbox for the future of recyclable soft robotic devices that require mechanical robustness, fast and reversible actuation as well as freedom in starting geometries.

## Conflict of interest

The authors declare no conflict of interest.

## Supporting information

As a service to our authors and readers, this journal provides supporting information supplied by the authors. Such materials are peer reviewed and may be re‐organized for online delivery, but are not copy‐edited or typeset. Technical support issues arising from supporting information (other than missing files) should be addressed to the authors.

Supplementary

Supplementary

Supplementary

Supplementary

Supplementary

Supplementary

## References

[1] M. Wei , Y. Gao , X. Li , M. J. Serpe , Polym. Chem. 2017, 8, 127–143.

[2] G. Stoychev , A. Kirillova , L. Ionov , Adv. Opt. Mater. 2019, 7, 1900067.

[3] A. Kirillova , L. Ionov , J. Mater. Chem. B 2019, 7, 1597–1624. 10.1039/c8tb02579g 32254904

[4a] H.-F. Lu , M. Wang , X.-M. Chen , B.-P. Lin , H. Yang , J. Am. Chem. Soc. 2019, 141, 14364–14369; 31429282 10.1021/jacs.9b06757

[4b] M. Pilz da Cunha , S. Ambergen , M. G. Debije , E. F. G. A. Homburg , J. M. J. den Toonder , A. P. H. J. Schenning , Adv. Sci. 2020, 1902842. 10.1002/advs.201902842 PMC7055549 32154076

[5] S. Nocentini , C. Parmeggiani , D. Martella , D. S. Wiersma , Adv. Opt. Mater. 2018, 6, 1–17.

[6] H. Zeng , P. Wasylczyk , D. S. Wiersma , A. Priimagi , Adv. Mater. 2018, 30, 1703554. 10.1002/adma.201703554 29067734

[7] B. Bhushan , T. Ma , T. Higashioji , J. Appl. Polym. Sci. 2002, 83, 2225–2244.

[8] H. K. Bisoyi , A. M. Urbas , Q. Li , Adv. Opt. Mater. 2018, 6, 1800458.

[9] H. K. Bisoyi , Q. Li , Chem. Rev. 2016, 116, 15089–15166. 27936632 10.1021/acs.chemrev.6b00415

[10] M. Yang , Z. Yuan , J. Liu , Z. Fang , L. Fang , D. Yu , Q. Li , Adv. Opt. Mater. 2019, 7, 1900069.

[11] Q. Li , Photoactive Functional Soft Materials, Wiley, Hoboken, 2019.

[12] S. Iamsaard , S. J. Aßhoff , B. Matt , T. Kudernac , J. J. L. M. Cornelissen , S. P. Fletcher , N. Katsonis , Nat. Chem. 2014, 6, 229–235. 24557138 10.1038/nchem.1859

[13] X. Pang , J. Lv , C. Zhu , L. Qin , Y. Yu , Adv. Mater. 2019, 31, 1904224. 10.1002/adma.201904224 31595576

[14] M. Wang , B. P. Lin , H. Yang , Nat. Commun. 2016, 7, 13981. 28004810 10.1038/ncomms13981 PMC5192217

[15] W. Wang , C. Li , M. Cho , S. H. Ahn , ACS Appl. Mater. Interfaces 2018, 10, 10419–10427. 29504740 10.1021/acsami.7b18079

[16] R. Yang , Y. Zhao , Angew. Chem. Int. Ed. 2017, 56, 14202–14206; 10.1002/anie.201709528 28960685

[17] Y. Dong , J. Wang , X. Guo , S. Yang , M. O. Ozen , P. Chen , X. Liu , W. Du , F. Xiao , U. Demirci , et al., Nat. Commun. 2019, 10, 4087. 31501430 10.1038/s41467-019-12044-5 PMC6733902

[18] J. M. Boothby , T. H. Ware , Soft Matter 2017, 13, 4349–4356. 28466922 10.1039/c7sm00541e

[19] K. M. Lee , T. J. Bunning , T. J. White , Adv. Mater. 2012, 24, 2839–2843. 22535595 10.1002/adma.201200374

[20] M. López-Valdeolivas , D. Liu , D. J. Broer , C. Sánchez-Somolinos , Macromol. Rapid Commun. 2018, 39, 3–9. 10.1002/marc.201700710 29210486

[21] A. S. Gladman , E. A. Matsumoto , R. G. Nuzzo , L. Mahadevan , J. A. Lewis , Nat. Mater. 2016, 15, 413–418. 26808461 10.1038/nmat4544

[22] C. P. Ambulo , J. J. Burroughs , J. M. Boothby , H. Kim , M. R. Shankar , T. H. Ware , ACS Appl. Mater. Interfaces 2017, 9, 37332–37339. 28967260 10.1021/acsami.7b11851

[23] B. Jin , H. Song , R. Jiang , J. Song , Q. Zhao , T. Xie , Sci. Adv. 2018, 10.1126/sciadv.aao3865. PMC5787381 29387791

[24] M. Barnes , R. Verduzco , Soft Matter 2019, 15, 870–879. 30628627 10.1039/c8sm02174k

[25] M. Behl , K. Kratz , J. Zotzmann , U. Nöchel , A. Lendlein , Adv. Mater. 2013, 25, 4466–4469. 23765645 10.1002/adma.201300880

[26] L. Yu , H. Shahsavan , G. Rivers , C. Zhang , P. Si , B. Zhao , Adv. Funct. Mater. 2018, 28, 1802809.

[27] M. K. McBride , A. M. Martinez , L. Cox , M. Alim , K. Childress , M. Beiswinger , M. Podgorski , B. T. Worrell , J. Killgore , C. N. Bowman , Sci. Adv. 2018, 10.1126/sciadv.aat4634. PMC6108565 30151428

[28] Z. Wang , Q. He , Y. Wang , S. Cai , Soft Matter 2019, 15, 2811–2816. 30882126 10.1039/c9sm00322c

[29] Y. Yang , E. M. Terentjev , Y. Zhang , Q. Chen , Y. Zhao , Y. Wei , Y. Ji , Angew. Chem. Int. Ed. 2019, 58, 17474–17479; 10.1002/anie.201911612 31529672

[30] J. J. Wie , K. M. Lee , T. J. White , Mol. Cryst. Liq. Cryst. 2014, 596, 113–121.

[31] K. M. Lee , H. Koerner , R. A. Vaia , T. J. Bunning , T. J. White , Soft Matter 2011, 7, 4318.

[32] M. Lahikainen , H. Zeng , A. Priimagi , Nat. Commun. 2018, 9, 4148. 30297774 10.1038/s41467-018-06647-7 PMC6175871

[33] T. Ube , K. Kawasaki , T. Ikeda , Adv. Mater. 2016, 28, 8212–8217. 27418031 10.1002/adma.201602745

[34] J. Hu , X. Li , Y. Ni , S. Ma , H. Yu , J. Mater. Chem. C 2018, 6, 10815–10821.

[35] R. C. P. Verpaalen , M. G. Debije , C. W. M. Bastiaansen , H. Halilović , T. A. P. Engels , A. P. H. J. Schenning , J. Mater. Chem. A 2018, 6, 17724–17729.

[36] Z. Yang , H. Peng , W. Wang , T. Liu , J. Appl. Polym. Sci. 2010, 116, 2658–2667.

[37] M. Dai , O. T. Picot , J. M. N. Verjans , L. T. De Haan , A. P. H. J. Schenning , T. Peijs , C. W. M. Bastiaansen , ACS Appl. Mater. Interfaces 2013, 5, 4945–4950. 23639415 10.1021/am400681z

[38] M. Pilz da Cunha , Y. Foelen , R. J. H. van Raak , J. N. Murphy , T. A. P. Engels , M. G. Debije , A. P. H. J. Schenning , Adv. Opt. Mater. 2019, 10.1002/adom.201801643.

[39] Z. Sekkat , Photo-Orientation by Photoisomerization, Woodhead Publishing Limited, Sawston, 2002.

[40] M. Pilz da Cunha , E. A. J. van Thoor , M. G. Debije , D. J. Broer , A. P. H. J. Schenning , J. Mater. Chem. C 2019, 7, 13502–13509.

[41] O. M. Tanchak , C. J. Barrett , Macromolecules 2005, 38, 10566–10570.

