# Supplementary material for: Liquid Crystal Networks on Thermoplastics: Reprogrammable Photo‐Responsive Actuators
Source: Angew Chem Int Ed Engl. 2020 Jan 30;59(11):4532–6. doi: 10.1002/anie.201915147 (PMC7065190; doi:10.1002/anie.201915147)
Supplement: Supplementary file 1 — Supplementary [file ANIE-59-4532-s001.pdf]

Angewandte Chemie
Eine Zeitschrift der Gesellschaft Deutscher Chemiker

Supporting Information

Liquid Crystal Networks on Thermoplastics: Reprogrammable Photo-Responsive Actuators

*Rob C. P. Verpaalen+, Marina Pilz da Cunha+, Tom A. P. Engels, Michael G. Debije, and Albert P. H. J. Schenning**

anie_201915147_sm_miscellaneous_information.pdf
anie_201915147_sm_Movie_S1.avi
anie_201915147_sm_Movie_S2.avi
anie_201915147_sm_Movie_S3.avi
anie_201915147_sm_Movie_S4.avi
anie_201915147_sm_Movie_S5.avi

Table of Contents

Table of Contents 1
Experimental Procedures 1
Figures 1
Author Contributions 4

Experimental Procedures

A light responsive coating was prepared by mixing 82 wt.% of covalent crosslinker 1,4-bis-[4-(6-acryloyloxyhexyloxy)benzoyloxy]-2-methylbenzene (RM1, Synthon), 16 wt.% of 4,4`-Bis[6-(acryloyloxy)hexyloxy]azobenzene (RM2, Synthon) and 2 wt.% of photo-initiator phenylbis(2,4,6-trimethylbenzoyl)phosphine oxide (PI, Merck). Tetoron® G2 Poly(ethylene terephthalate) (PET) substrates were kindly provided by Teijin.

Liquid crystal network (LCN) films of 30 x 30 mm^2 were prepared by filling 20 µm gap liquid crystal (LC) cells coated with rubbed polyimide alignment layers. The alignment of the LCs was preserved by UV photo polymerization (20 mW/cm^2 for 600 seconds under nitrogen) at 85 °C followed by a post-curing step, 20 minutes at 130 °C. A cut-off filter ($\lambda \geq 400$ nm, Thorlabs FGL400S) was used during photo polymerization. LCN films were carefully removed from the polyimide-coated glass cell.

Oriented PET substrates were rinsed by ultra-sonication in isopropanol, dried with compressed air, UV-ozone treated for 60 minutes and subsequently LCs were spray applied from xylene (1:5 by weight) employing an AMI 200 airbrush. Using nitrogen as carrier gas at 1 bar, a ~ 4 µm LCN coating was applied onto 12 µm PET substrates and UV photo polymerized as previously described.[26,28]

Anisotropic mechanical properties of freestanding polymer films were measured on a TA instruments Q800 dynamic mechanical analyser (DMA). Elastic moduli (*E'*) and *tan δ* values were determined for 10 x 5.3 mm^2 films at a 1 Hz single frequency and 10 µm amplitude. Controlled force experiments were conducted to analyse the thermal expansion of oriented films.

Collimated 365 and 455 nm light emitting diodes (Thorlabs M365L2 and M455L3) were employed to investigate light triggered actuation in LCN/PET bilayers. All experiments were performed at ambient conditions. Photographs were taken utilizing an Olympus E-M10 Mark III and accordingly analysed per frame using ImageJ open access software.

Figures

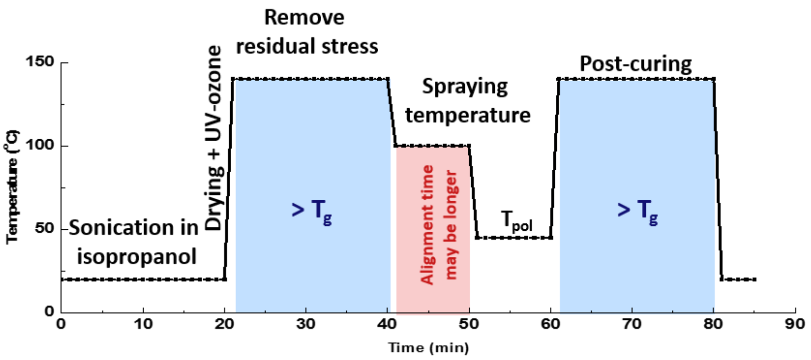

Figure S1. Graphical representation of the bilayer preparation procedure.

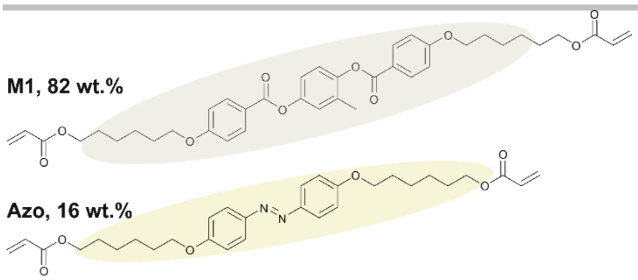

Figure S2. Molecular structures of the LC mesogens in the liquid crystal mixture.

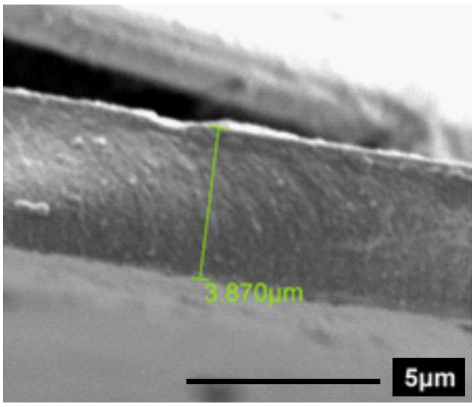

Figure S3. SEM cross-section of the cryo-fractured LCN coating.

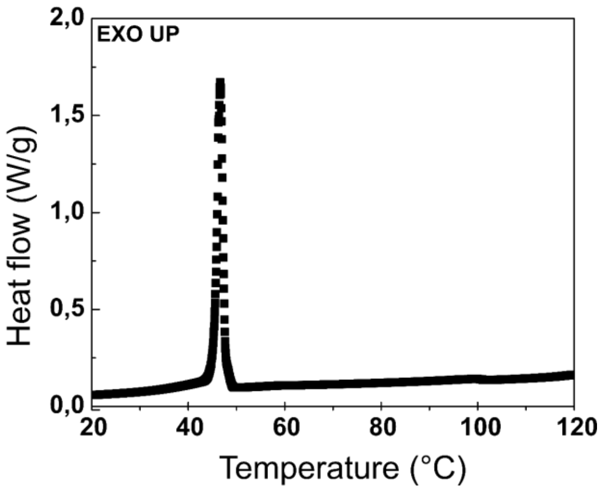

Figure S4. Dynamic scanning calorimetry of the liquid crystalline mixture, second cycle, cooling, 7 °C/min.

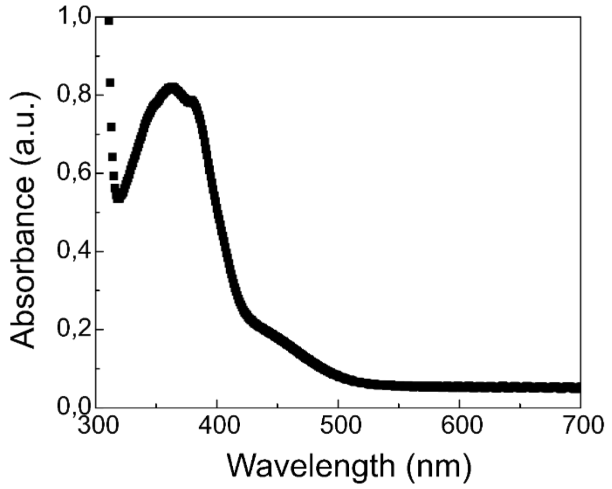

Figure S5. Absorbance spectrum of the bilayer actuator prior to illumination.

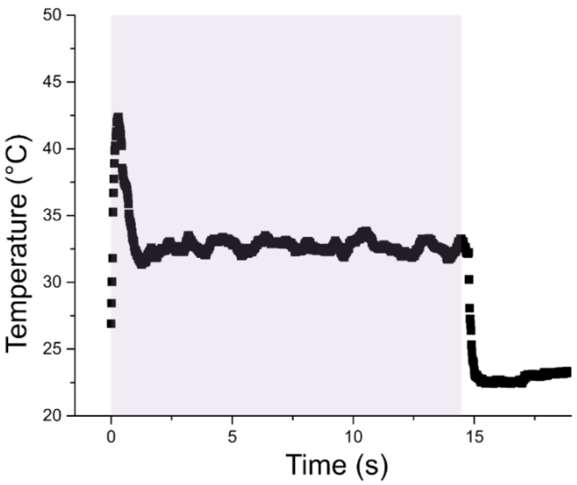

Figure S6. Surface temperature profile of the bilayer actuator upon illumination with 365 nm light with 170 mW/cm^2 intensity. The violet box represents the time in which the UV light is turned on.

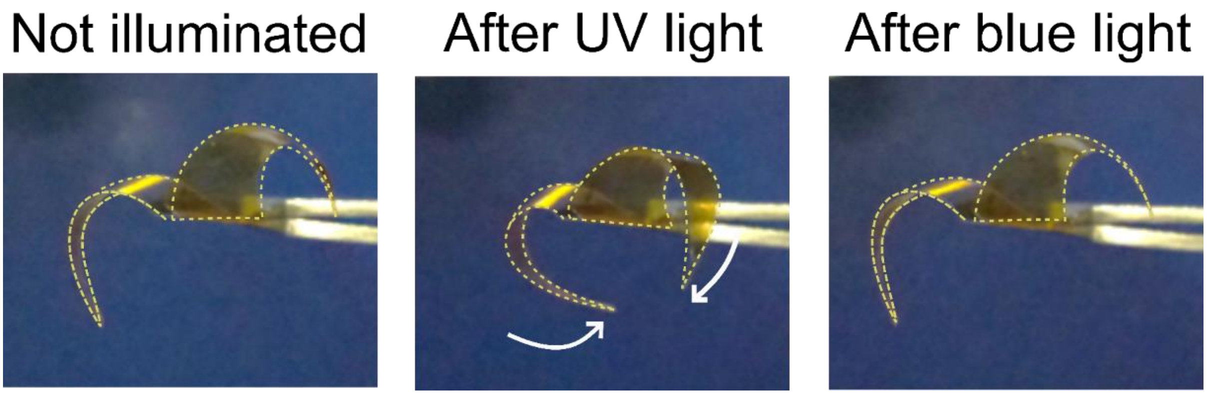

Figure S7. Bilayer actuator moulded into an origami-like bird shape. The LCN coated side in on the inside of the wigs. UV light irradiation of the wings causes their downwards bend. Blue light causes the upward motion.

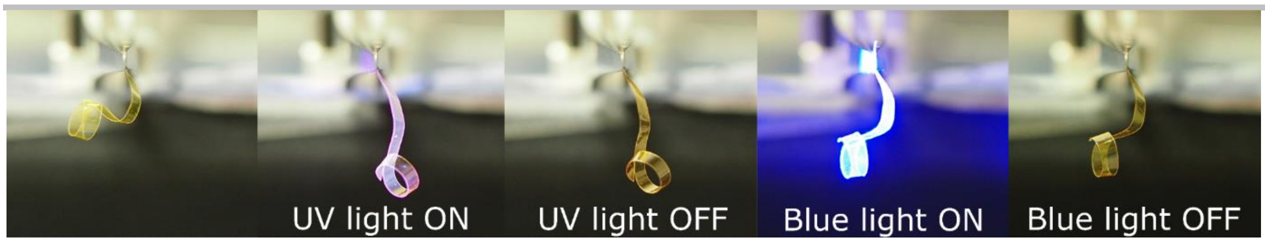

Figure S8. Light triggered deformation of the bilayer actuator moulded into an arbitrary shape.

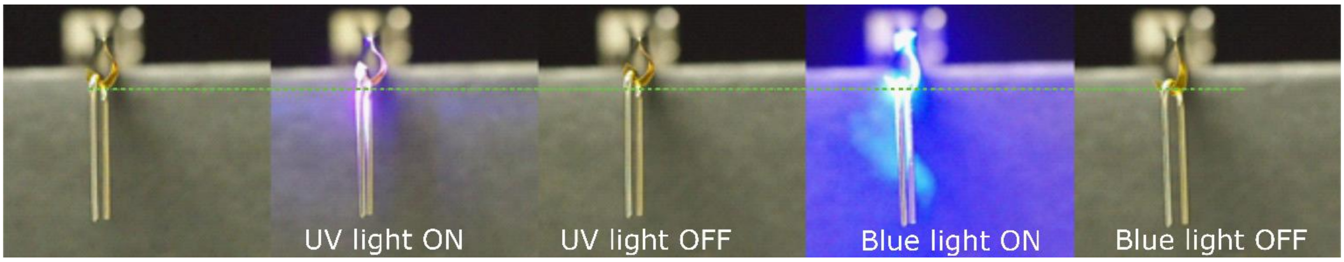

Figure S9. A demonstration of the pre-deformed bilayer (weighing 1 mg and with the following dimensions: 7 x 3 x 0.016 mm^3), lifting a paper clip weighing 140 mg upon UV light exposure. A green line to guide the eye is drawn to show the extent of lifting performed by the bilayer

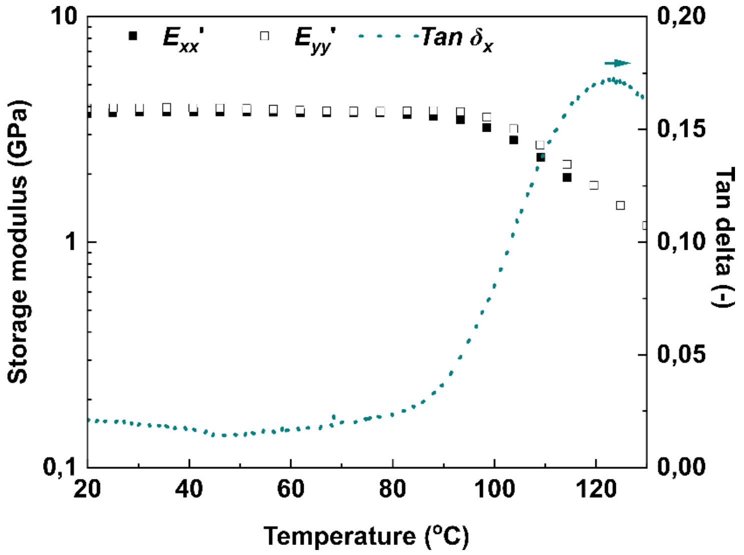

Figure S10. Dynamic mechanical analysis of biaxially stretched PET demonstrates similar storage moduli curves in both x and y directions.

Author Contributions

MD and AS conceived the research. RV and MP made the films, characterized the mechanical properties and measured their photo-response. All authors contributed to writing the document.
